# Pelvic floor muscles after prostate radiation therapy: morpho-functional assessment by magnetic resonance imaging, surface electromyography and digital anal palpation

**DOI:** 10.1590/S1677-5538.IBJU.2019.0765

**Published:** 2020-11-18

**Authors:** Aline Moreira Ribeiro, Larissa Guerra Nammur, Elaine Cristine Lemes Mateus-Vasconcelos, Cristine Homsi Jorge Ferreira, Valdair Francisco Muglia, Harley Francisco de Oliveira

**Affiliations:** 1 Curso de Educação Física Departamento de Ciências da Saúde Campo BeloMG Brasil Departamento de Ciências da Saúde, Curso de Educação Física, Centro Mineiro do Ensino Superior, Campo Belo, MG, Brasil; 2 Universidade Federal de Uberlândia Faculdade de Fisioterapia e Educação Física UberlândiaMG Brasil Faculdade de Fisioterapia e Educação Física, Universidade Federal de Uberlândia, Uberlândia, MG, Brasil; 3 Centro Universitário Barão de Mauá Departamento de Fisioterapia Ribeirão PretoSP Brasil Departamento de Fisioterapia, Centro Universitário Barão de Mauá, Ribeirão Preto, SP, Brasil; 4 Universidade de São Paulo Faculdade de Medicina de Ribeirão Preto Departamento de Biomecânica, Medicina e Reabilitação do Sistema Locomotor Ribeirão PretoSP Brasil Departamento de Biomecânica, Medicina e Reabilitação do Sistema Locomotor, Faculdade de Medicina de Ribeirão Preto, Universidade de São Paulo, Ribeirão Preto, SP, Brasil; 5 Universidade de São Paulo Faculdade de Medicina de Ribeirão Preto Departamento de Clínica Médica Ribeirão PretoSP Brasil Departamento de Clínica Médica, Divisão de Radiologia, Faculdade de Medicina de Ribeirão Preto, Universidade de São Paulo, Ribeirão Preto, SP, Brasil; 6 Universidade de São Paulo Faculdade de Medicina de Ribeirão Preto Departamento de Clínica Médica Ribeirão PretoSP Brasil Departamento de Clínica Médica, Divisão de Radioterapia, Faculdade de Medicina de Ribeirão Preto, Universidade de São Paulo, Ribeirão Preto, SP, Brasil

**Keywords:** Pelvic Floor, Prostatic Neoplasms, Radiotherapy

## Abstract

**Aim:**

To evaluate the radiotherapy (RT) effect in the pelvic floor muscles (PFM) function in men with prostate cancer (PC).

**Materials and Methods:**

A cross-sectional study included three groups of patients with PC and RT indication: 1) Pre-RT group: evaluated before the beginning of RT; 2) Acute group: evaluated between six months and one year after RT; 3) Late Group: evaluated between two and a half years and four years post-RT. PFM assessment was divided into: a) functional assessment through the digital anal palpation (Modified Oxford Scale) and surface electromyography (sEMG) with anal probe; b) anatomical assessment by pelvic magnetic resonance imaging (MRI) with thickness measurements of levator ani muscle and pelvic specific parameters at rest and under Valsalva maneuver. We used Student t test, considering as significant p <0.05.

**Results:**

Thirty-three men were assessed: Pre-RT (n=12); Acute (n=10) and Late (n=11) groups. PFM functional assessment showed Late group with lower electromyographic activity, especially in the sustained contractions when compared to the Pre-RT (p=0.003) and Acute groups (p=0.006). There was no significant difference between groups in MRI.

**Conclusion:**

PFM functional assessment showed a decrease in sEMG activity in the Late group post-RT. Most of the sample (72.7%) did not know how to actively contract the PFM or had a weak voluntary contraction when assessed by digital anal palpation. Also, these patients presented higher prevalence of pelvic complaints. No changes were observed in the morpho-functional parameters evaluated by MRI, except the measurement of the membranous urethra length when comparing Pre-RT Group and Acute and Late Groups.

## INTRODUCTION

The treatment for Prostate Cancer (PC) must be individualized, considering aspects such as age, tumour stage and grade, comorbidities, life expectancy, patient wishes and available technical resources (1). Considering therapy options, radiotherapy (RT) has taken a key role because of the significant rates of local disease control and reduction of adverse effects (2).

Radiation effects can be divided into acute and late effects. Acute damage is quickly repaired due to the fast proliferation of stem cells and can be completely reversible. While late effects appear after months or years and may regress, but without complete repair (3). However, literature data analysing if pelvic floor muscles (PFM) function may or may not be affected by prostate irradiation is scarce.

Cumulative doses of 45Gy appear to produce detectable muscle damage within three years post-RT in patients with good overall health. However, in patients suffering from cachexia or other debilitating diseases, muscle necrosis was constantly observed after exposure with 20Gy. Muscle fibrosis is a common effect of irradiation on skeletal muscle. However, the precise mechanism of RT effects on neuromuscular junction is unknown. Temporary changes in K^+^ and Na^+^ membrane permeability, the release of pharmacologically active substances such as serotonin and acetylcholine and the interaction between lipoproteins and free radicals may contribute to post-RT synaptic changes. Changes in Na^+^ and K^+^ membrane permeability influence Na^+^/K^+^ pump activity leading to changes in muscle excitability and contractility. However, the alteration mechanism of neuromuscular junction has not been completely clarified, requiring further investigation. Little is known about the mechanism of muscle adaptation after irradiation (3).

It is not known whether radiation can initiate important anatomical and/or functional changes in PFM, manifesting clinically as pelvic dysfunctions. Then, the paucity of data in this issue was our motivation for this research, focusing on the PFM functional assessment in men who underwent RT for PC. The aim of this study was to evaluate the RT effect in the PFM function in men treated for PC.

## MATERIALS AND METHODS

### Study design

This cross-sectional study was conducted at Imaging Sciences and Medical Physics Center of the University of São Paulo. Patients were consecutively selected according to chronological order of treatment time in the Radiotherapy Section. The recruitment of patients happened in August, September and October 2013. Data collection happened between August 2013 and April 2014. Those who agreed to participate in the study had to sign an Informed Consent Term.

Inclusion criteria comprised men who were diagnosed with PC confirmed by biopsy with indication for RT concomitant or not with other cancer treatments: radical prostatectomy (RP) and androgen deprivation therapy (ADT); age over 45 years and that have agreed to participate. Exclusion criteria were anatomical changes in the perineal region due surgery or trauma sequelae, neurological disease, acquired immunodeficiency syndrome, RT not completed, prior PFM training, use of cardiac pacemaker or other devices that prevent completion of the study protocol.

Patients were divided into three groups:

1. Pre-RT: assessed before the beginning of RT;
2. Acute: six months to one year post-RT;
3. Late: two and a half to four years post-RT.
  All patients were submitted to the same tests and exams, made by the same physiotherapist, except for MRI examinations.

### Pelvic floor assessment

The patients had notions of PFM anatomy and function through illustrative figures, in order to improve their understanding about the location of these muscles, helping them to contract properly the PFM. Digital anal palpation was primarily performed before the initial evaluation of the PFM, in order to confirm that patients were able to contract effectively these muscles.

The patients were positioned in the left lateral decubitus. The PFM voluntary contraction evaluation was done by digital anal palpation with verbal commands to specific movements as if it were “hold pee” or “retain flatus”. The subject was instructed about the adequate muscle contraction, preventing the simultaneous contraction of accessories muscles. The grading of performed contraction was made according to Modified Oxford Scale (4).

In the same position, PFM activity was collected by surface electromyography (sEMG) (Electromyograph Miotool Uro, Miotec^®^ Biomedical Equipment LTD, Rio Grande do Sul, Brazil) with a specific anal probe lubricated with water-based gel and inserted into the anal canal. The reference electrode was placed in the right anterior superior iliac spine. The subject was instructed to perform three maximal voluntary contractions (MVC) of two seconds succeeded by a rest of 10 seconds. The highest value was used for normalization. The sEMG protocol used was described by Glazer et al. (5): 60 seconds of initial rest, 5 fast contractions with 2 seconds of lift preceded by a rest period of 10 seconds each, 5 contractions sustained for 10 seconds preceded by a rest period of 10 seconds each, single contraction sustained for 60 seconds preceded and succeeded by a rest period of 10 seconds, 60 seconds of final rest. For data analysis, we used gain of 500, high-pass filter of 20Hz and down-pass filters of 500Hz. The normalization of the sEMG data followed the recommendations proposed (6, 7), attributing the value of 100% for MVC.

### MRI protocol

**Figure 1:**
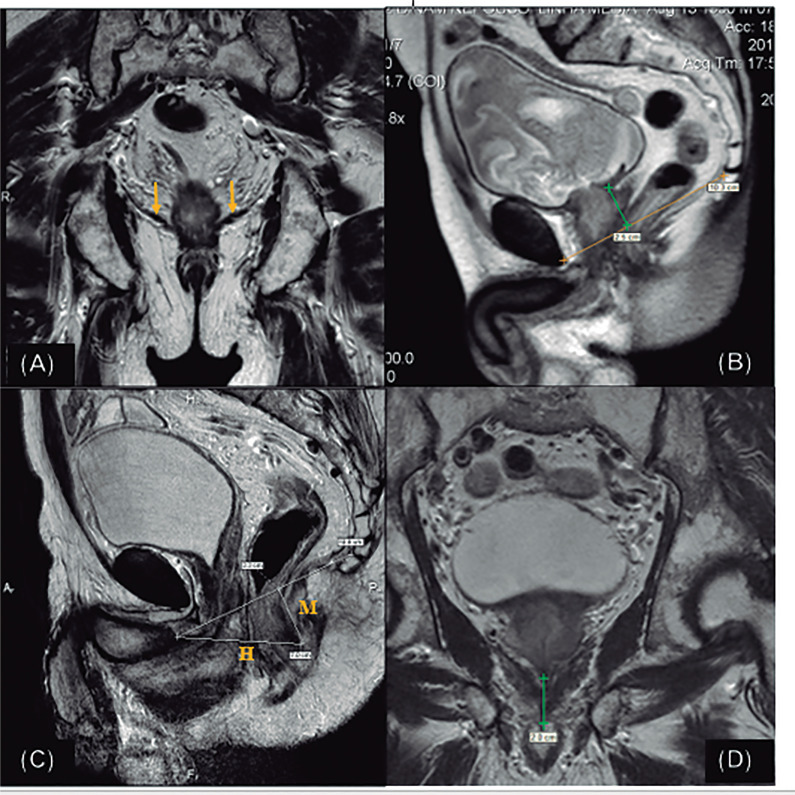
MRI standard measurements - A): Axial T2-weighted MR image shows LA thickness; B): Sagittal T2-weighted MR image shows the distance bladder to pubococcigeal line (DBPCL); C): Sagittal T2-weighted MR image shows the H and M lines; D): Coronal T2-weighted MR image shows the membranous urethra length (MU)

For the PFM anatomical assessment, we used a 1.5 Tesla MRI scanner, Achieva (Philips Medical Systems, Best, The Netherlands), with a six-channel, phased-array pelvic coil and the images were acquired in the axial, coronal and sagittal planes, T1 and T2-weighted and dynamic images (at rest and during Valsalva maneuver). Standard measurements are described below:

1. Levator ani muscle (LA) thickness: By drawing a horizontal line between the ischial tuberosity as the reference for measuring right and left portions of LA (Figure-1A).
2. Distance bladder to pubococcigeal line (DBPCL): As understood from the bladder floor to pubococcigeal line (PCL) at rest (DBPCL-R) and during the Valsalva maneuver (DBPCL-V) (Figure-1B).
3. H Line (HL): Distance from the bottom of the pubis to the posterior anorectal junction at rest (HL-R) and during the Valsalva maneuver (HL-V) (Figure-1C).
4. M Line (ML): line perpendicular to PLC measured from the posterior anorectal junction in relation to the PCL at rest (ML-R) and during the Valsalva maneuver (ML-V) (Figure-1C).
5. Membranous urethra (MU) lenght (Figure-1D).

The same radiologist with large experience in uroradiology performed all measurements.

### RT protocol

Patients underwent Three-dimensional Conformal Radiation Therapy (3D-CRT) or Intensity Modulated Radiation Therapy (IMRT) in prostate. The fractionation was 2.0Gy/fraction (day-fraction, five days a week) using total doses for low-risk of 76 to 78Gy in 38-39 fractions. For intermediate and high-risk, in the first stage of treatment was prescribed 52 to 56Gy in 26 to 28 fractions, and in the second stage, 20 to 26Gy dose in 10 to 13 fractions, also 2.0Gy/fraction (one fraction, five days a week).

### Pelvic symptoms

The patient's medical history, their sociodemographic and personal data, as comorbidities, age, weight, height, schooling, race, marital status and health habits were obtained. Urological, anorectal, and sexual history were obtained through medical records analysis and confirmed with patients during the interview.

#### Statistical Analysis

Initially, an exploratory data analysis was performed considering the central position measures (mean and median) and the dispersion measures (standard deviation). Student's t-test was applied to verify differences between groups, regarding the quantitative variables. The analyzes were implemented in the SAS program version 9.4. The significance level adopted for all tests was 5%.

### Ethical aspects

This research was approved by the Research Ethics Committee of University of São Paulo (n° 3014/2013).

## RESULTS

**Figure 2:**
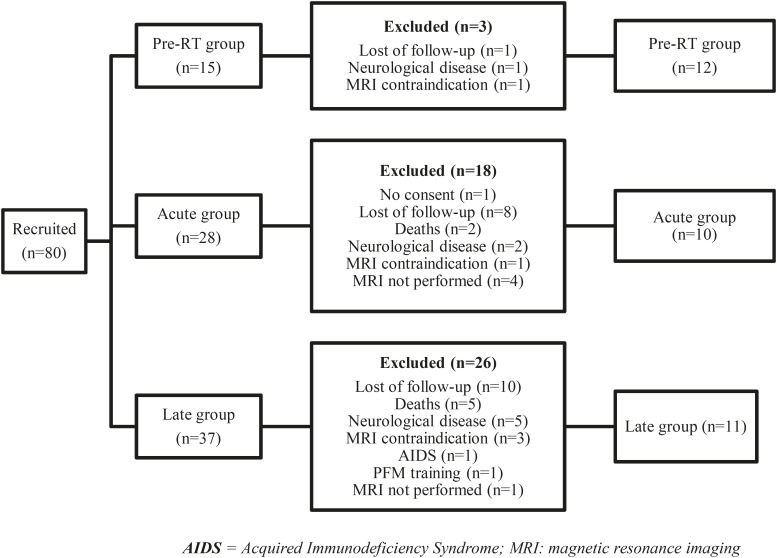
AIDS - Acquired Immunodeficiency Syndrome

Thirty-three patients were enrolled, according to the flowchart below demonstrated in the Figure-2.

The average age was 71.4 (1.9) (61-81 years), 67.5 (3.1) (51-79 years) and 70.5 (2.2) (59-81 years) to pre-RT, acute and late groups, respectively. The sociodemographic and personal data are shown in Table-1. There was no significant difference related to age and body mass index between the groups (p=0.4).

**Table 1:** Sociodemographic and personal data [mean±SD; n (%); (range)]

		Pre-RT group (n=12)	Acute group (n=10)	Late group (n=11)
Age (years)		71.4±1.9	67.5±3.1	70.5±2.2
		(61-81 years)	(51-79 years)	(59-81 years)
BMI (kg/m2)		27.9±1.7	29.8±1.8	26.3±1.3
**Schooling**				
	Illiterate	4 (33.3)	1 (10)	2 (18.2)
	Primary school	7 (58.3)	7 (70)	7 (63.6)
	Middle school	1 (8.3)	1 (10)	0 (0)
	Technical school	0 (0)	1 (10)	0 (0)
	Higher education	0 (0)	0 (0)	2 (18.2)
**Marital status**				
	Single	0 (0)	1 (10)	0 (0)
	Married	9 (75)	9 (90)	6 (54.5)
	Divorced	2 (16.7)	0 (0)	4 (36.4)
	Widower	1 (8.3)	0 (0)	1 (9.1)
**Color**				
	White	9 (75)	8 (80)	8 (72.7)
	Black	3 (25)	2 (20)	3 (27.3)
	Yellow	0 (0)	0 (0)	0 (0)
**Comorbidities**				
	Hypertension	8 (66.7)	6 (60)	5 (45.5)
	Diabetes mellitus	2 (16.7)	1 (10)	2 (18.2)
**Health habits**				
	Sedentary lifestyle	10 (83.3)	5 (50)	11 (100)
	Alcoholism	1 (8.3)	3 (30)	6 (54.5)
	Smoking	3 (25)	1 (10)	2 (18.2)

**BMI** = body mass index.

Pre-RT group showed the greatest history of high-risk PC (75%). Forty percent of the patients in the Acute, 27.3% in the Late and 8.3% in the Pre-RT groups had RP. ADT was most prevalent in the Pre-RT group (91.7%). There was no significant difference in dosimetry and number of RT fractions (p=0.3).

The PFM functional assessment by digital anal palpation demonstrated that 24 patients (72.7%) were classified with grades 0, 1 and 2 according to Modified Oxford Scale, which indicates that most of the men did not know how to actively contract the PFM or had a weak voluntary contraction.

The sEMG analysis is shown in Table-2 and values are presented already normalized. The measurements assessed by MRI are described in Table-3.

**Table 2:** Electromyographic data for the three groups [mean (SD)]

sEMG		Pre-RT vs. Acute (n=12) vs. (n=10)		p	Pre-RT vs. Late (n=12) vs. (n=11)		p	Acute vs. Late (n=10) vs. (n=11)		p
**Sustained contractions**										
	Contraction 1	19.1 (3.2)	17.8 (2.9)	0.6	19.1 (3.2)	17.6 (3.1)	0.3	17.8 (2.9)	17.6 (3.1)	0.6
	Contraction 2	22.1 (3.7)	21.2 (3.0)		22.1 (3.7)	21.5 (3.2)		21.2 (3.0)	21.5 (3.2)	
	Contraction 3	22.4 (4.3)	22.9 (3.5)		22.4 (4.3)	20.2 (2.8)		22.9 (3.5)	20.2 (2.8)	
	Contraction 4	23.0 (5.0)	23.2 (3.1)		23.0 (5.0)	21.9 (3.6)		23.2 (3.1)	21.9 (3.6)	
	Contraction 5	22.1 (5.1)	20.6 (2.9)		22.1 (5.1)	21.4 (3.6)		20.6 (2.9)	21.4 (3.6)	
**Fast contractions**										
	Contraction 1	19.5 (3.6)	16.8 (2.1)	0.2	19.5 (3.6)	14.5 (1.8)	0.003	16.8 (2.1)	14.5 (1.8)	0.006
	Contraction 2	18.3 (3.9)	15.2 (2.1)		18.3 (3.9)	13.6 (1.9)		15.2 (2.1)	13.6 (1.9)	
	Contraction 3	19.2 (4.6)	17.5 (2.4)		19.2 (4.6)	13.7 (1.8)		17.5 (2.4)	13.7 (1.8)	
	Contraction 4	18.5 (4.0)	17.0 (2.3)		18.5 (4.0)	13.2 (1.7)		17.0 (2.3)	13.2 (1.7)	
	Contraction 5	17.4 (3.8)	16.3 (2.1)		17.4 (3.8)	12.3 (1.7)		16.3 (2.1)	12.3 (1.7)	
	Single contraction (60s)	12.9 (2.2)	13.4 (1.8)		12.9 (2.2)	9.8 (1.2)		13.4 (1.8)	9.8 (1.2)	

**RT** = radiotherapy

**Table 3:** Anatomical parameters measured by MRI [mean (SD)]

MRI	Pre-RT vs. Acute (n=12) vs. (n=10)		p	Pre-RT vs. Late (n=12) vs. (n=11)		p	Acute vs. Late (n=10) vs. (n=11)		p
LA-R (mm)	21.4 (4.2)	25.5 (2.9)	0.5	21.4 (4.2)	30.5 (3.0)	0.09	25.5 (2.9)	30.5 (3.0)	0.2
LA-L (mm)	22.6 (4.1)	28.4 (4.1)	0.3	22.6 (4.1)	28.3 (4.7)	0.4	28.4 (4.1)	28.3 (4.7)	1.0
DBPCL-R (mm)	27.2 (4.6)	24.6 (4.3)	0.7	27.2 (4.6)	23.5 (3.7)	0.5	24.6 (4.3)	23.5 (3.7)	0.9
DBPCL-V (mm)	18.3 (5.4)	18.0 (3.4)	1.0	18.3 (5.4)	16.5 (3.5)	0.8	18.0 (3.4)	16.5 (3.5)	0.8
DBPCL-R (-) DBPCL-V (mm)	9.3 (2.4)	6.6 (1.4)	0.4	9.3 (2.4)	7.0 (1.1)	0.4	6.6 (1.4)	7.0 (1.1)	0.8
HL-R (mm)	60.3 (2.1)	62.7 (3.4)	0.5	60.3 (2.1)	62.3 (2.7)	0.5	62.7 (3.4)	62.3 (2.7)	0.9
HL-V (mm)	59.9 (3.3)	65.0 (3.6)	0.3	59.9 (3.3)	65.6 (2.4)	0.2	65.0 (3.6)	65.6 (2.4)	0.9
HL-R (-) HL-V (mm)	0.3 (2.2)	-2.3 (0.7)	0.5	0.3 (2.2)	-3.3 (1.1)	0.3	-2.3 (0.7)	-3.3 (1.1)	0.7
ML-R (mm)	29.7 (3.7)	23.9 (2.9)	0.2	29.7 (3.7)	24.8 (1.5)	0.3	23.9 (2.9)	24.8 (1.5)	0.8
ML-V (mm)	31.8 (1.9)	28.7 (2.7)	0.4	31.8 (1.9)	30.2 (2.2)	0.6	28.7 (2.7)	30.2 (2.2)	0.7
ML-R (-) ML-V (mm)	-2.1 (2.3)	-4.8 (1.3)	0.5	-2.1 (2.3)	-5.4 (1.5)	0.4	-4.8 (1.3)	-5.4 (1.5)	0.8
MU (mm)	12.1 (1.0)	15.7 (1.1)	0.02	12.1 (1.0)	14.9 (0.8)	0.04	15.7 (1.1)	14.9 (0.8)	0.5

**LA-R** = levator ani muscle right side; **LA-L** = levator ani muscle left side; **DBPCL-R** = distance bladder to pubococcigeal line in rest; **DBPCL-V** = distance bladder to pubococcigeal line in Valsalva; **DBPCL-R (-) DBPCL-V** = difference between DBPCL-R and DBPCL-V; **HL-R** = H line in rest; **HL-V** = H line in Valsalva; **HL-R (-) HL-V** = difference between HL-R and HL-V; **ML-R** = M line in rest; **ML-V** = M line in Valsalva; **ML-R (-) ML-V** = difference between ML-R and ML-V; **MU** - membranous urethra.

**Figure 3:**
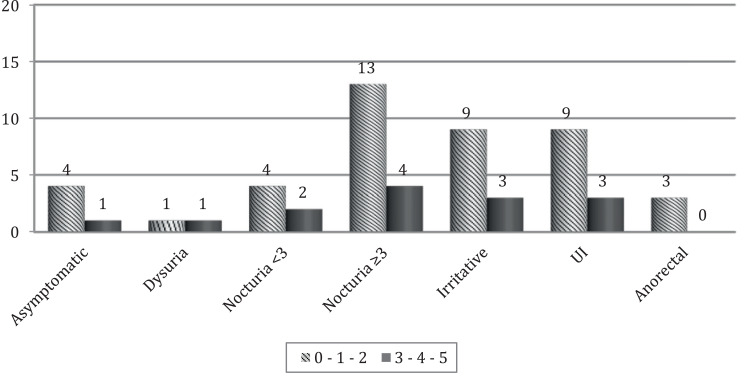
Distribution of pelvic complaints according the functional evaluation of PFM by digital palpation (n)

The distribution of urinary and anorectal complaints according the functional evaluation of PFM by digital palpation is described in Figure-3. Patients classified with grades 0, 1 and 2 presented higher prevalence of pelvic complaints. The values presented are expressed as number of patients (n).

## DISCUSSION

There is an evident bias in medical literature in favour of females when studying PFM and its related disorders. The higher prevalence of complaints in women, compared to men may provide a rationale for this scenario. However, male PFM dysfunctions, although less frequent, also cause relevant negative effects on quality of life (QoL) (8).

While sharing the same functions on both genders, it is known that the male PFM possess unique functions. The PFM play an important role, supporting the contents of the abdomen, helping in the maintenance of orthostatic posture and it is crucial in the maintenance of urinary and faecal continence, in both genders. However, in men, it is crucial in the achievement and maintenance of penile erection and in the ejaculation process (8).

Smeenk et al. (9) investigated the relationship between RT dose-volume in PFM and anorectal complaints in PC. Internal and external anal sphincter (EAE), puborectalis (PUR) and LA muscles, were retrospectively outlined, beyond rectal and anal walls when planning RT using CT. Their results showed the total amount of radiation in PFM was about three times greater than for anal wall. The PUR was exposed to the highest dose used in RT, whereas EAE received the lower dose. Various anal and rectal dose parameters, as well as for all PFM were associated with faecal urgency, while anal incontinence was mainly associated with high doses to EAE and PUR.

Although Smeenk et al. (9) had done a pioneer study assessing the RT effect in PFM, they did not evaluate the functional and anatomical changes these muscles may have suffered. However, as the PFM are striated skeletal muscles and have the same composition of muscle fibbers than other skeletal striated muscles, studies related to radiation from other areas can help us to understand the effect of radiation on function and muscular anatomy.

Shamley et al. (10) and Tedla et al. (11) assessed functional changes in muscle fibbers after RT. The latter one studied the RT impact in the soft tissues of the larynx and found no significant difference related to the amount of muscle fibrosis, but qualitative changes, such as changes in the organization of muscle fibbers (11). Shamley et al. (10) described the level of electromyographic activity and muscle size in the shoulders of patients irradiated for breast cancer. Three of the four muscles of the treated breast demonstrated lower electromyographic activity. Upper trapezius muscle showed great loss of electromyographic activity. The loss of this activity was significantly associated with decrease in shoulder function. There was a significant decrease in muscle size of the major and minor pectoralis by MRI.

The results found by Shamley et al. (10) in breast cancer corroborate our data. Our sEMG findings showed significant differences in the evaluation of sustained contractions on the Late group (p=0.003 and p=0.006). Both studies indicate a tendency to muscle function loss when relevant muscle fibbers damage occurs. This inference can be made for our study because PFM dysfunctions may be related to the onset and/or severity of urinary and anorectal complaints. Many of the PFM disorders are caused by inadequate motor recruitment patterns, muscle weakness or inadequate muscle coordination which could increase the risk of urinary loss during efforts or the incapacity to postpone micturition until an appropriate location is found (12).

In our study, functional changes detected by sEMG did not show correlation with anatomical changes evaluated by MRI. However, the majority of the studies provide MRI standard values of reference for the female PFM. MRI is an excellent tool to understand the complex anatomy of the PFM and to assess pelvic floor disorders, since it enables static and dynamic imaging of the pelvic floor. Using static T2-weighted sequences the morphology of the pelvic floor can be visualized in great detail. A rapid half-Fourier T2-weighted, balanced steady state free precession, or gradient-recalled echo sequence are used to obtain sagittal images while the patient is at rest, during pelvic squeeze, pelvic strain and to document the evacuation process. On these images the radiologist identifies PCL (13).

The accuracy of total pelvic floor ultrasound for anatomical abnormalities when compared with defecatory MRI was assessed by Hainsworth at al. (14). They found that sensitivity and specificity of total pelvic floor ultrasound were 81% and 33% for rectocele, 60% and 91% for intussusception, 65% and 80% for enterocele and 65% and 84% for cystocele when compared with defecatory MRI.

At rest, the base of the bladder is at or above the PCL. During the Valsalva maneuver, PFM tend to relax down normally less than three inches below the PCL (15). Confronting this reference value to our results, the Acute and Late groups had lower values between DBPCL-R and DBPCL-V, demonstrating a greater bladder descent during effort - not significant however within the reference standards cited by Goh et al. (15). These authors analyzed 50 healthy volunteers (25 men) and found a variation of 7.0mm in men, within the range found in our study.

Along with the measurements related to PCL, there is a rating system commonly used in other pelvic disorders by MRI: the HMO system (H and M lines and pelvic organ prolapse - POP) (16). This classification system is used to quantify the degree of POP, indicating its muscle insufficiency during efforts. The values of H and M lines have a direct correlation with the degree of muscle weakness and PFM descent during Valsalva (17). However, we reiterate that most studies using this classification system evaluated only women. Consequently, its use as a standard reference for men is questionable, even though it was the only available reference. The HMO system classifies the PFM failure in three categories, considering the M and H lines: mild, moderate and severe (16). There was a tendency of greater descent of PFM in the Late group compared to the others, which may be explained by the worst muscle function assessed by sEMG, but the difference was not significant.

The MU length is another parameter associated with recovery of urinary continence in the first year after RP (18). Coakley et al. (19), analyzing data from 211 men submitted to RP, have shown that men with urethral length greater than 12mm showed complete urinary continence in one year, while patients with urethral length less than 12mm showed variable frequency of urinary incontinence. In our study, we evaluated the MU and the values coincided the cut-off value proposed by Coakley et al. (19). However, RT contributes to urethral stricture because of the vascular damage, ischemia, and fibrosis (20).

For measurement of LA thickness, there are no reference values to confront our findings. There was no difference between the groups, however pre-RT group showed the smallest LA thickness for both sides. This may be due to the high number of patients undergoing ADT.

Our study has some limitations. The main one is the inclusion of patients submitted to RP and ADT. Sphincter function after RP relies on the integrity of the external urethral sphincter. After removal of the internal urethral sphincter during RP, the intravesical resistance is maintained by the external urethral sphincter action (21). A few studies including PFM training in the management of urinary and sexual complaints provided scarce information of the PFM function before and after RP. Therefore, functional changes after RP are not fully understood. Studies indicated that ADT has significant impact on musculoskeletal status by increasing the percentage of fat mass and decreasing fat-free mass (22), but there is no data about the relationship “PFM and ADT”.

The short period of observation in this study precludes follow-up of the same patients before and after RT. However, although most studies with men include only patients submitted to RP (therefore excluding those sent to RT), we decided to include this subset of patients to gather information. The lack of studies in this field led us to give an overview about the situation of these patients. There are no data about the PFM behavior in this sample. Thus, to this end, a cross-sectional study fulfils its objectives. It is important to notice that this is a cross-sectional study and we are unable to make predictions. Another point is that a lack of studies focusing on male PFM makes analysis and comparison of data (both for sEMG and for MRI) a difficult task, as there are no standard of references for many parameters used at PFM functional assessment, regardless of the method used. Despite the limitations, our main motivation with this research was to outline the scenario in which these patients are inserted, to analyze its context and to stimulate a more comprehensive investigation of the matter. In PC, the studies include prostatectomized men, while RT is an exclusion criterion of subjects. Understanding the adverse effects of RT in the PC treatment may help to understand the impact of treatment on patients and to stimulate the search for strategies to prevent or minimize these complaints. Physical therapy has much to contribute for the improvement of PFM function in these patients, with consequent better QoL and functionality to the patients. However, there are few studies focused on the male PFM.

## CONCLUSION

PFM functional assessment showed a decrease in sEMG activity in the Late group post-RT. Most of the sample (72.7%) did not know how to actively contract the PFM or had a weak voluntary contraction when assessed by digital anal palpation. Also, these patients presented higher prevalence of pelvic complaints. No changes were observed in the morpho-functional parameters evaluated by MRI, except the measurement of the membranous urethra length when comparing Pre-RT Group with Acute and Late Groups.
